# The genetic structure and diversity of smallholder dairy cattle in Rwanda

**DOI:** 10.1186/s12863-025-01323-4

**Published:** 2025-05-27

**Authors:** Oluyinka Opoola, Felicien Shumbusho, Innocent Rwamuhizi, Isidore Houaga, David Harvey, David Hambrook, Kellie Watson, Mizeck G. G. Chagunda, Raphael Mrode, Appolinaire Djikeng

**Affiliations:** 1 Global Academy of Agriculture and Food Systems (GAAFS) and the Royal (Dick) School of Veterinary Studies (RDSVS), University of Edinburgh, Easter Bush Campus, Edinburgh, UK; 2 Rwanda Agriculture and Animal Resources Development Board (RAB), Kigali, Rwanda; 3 Centre for Tropical Livestock Genetics and Health (CTLGH), The Roslin Institute, University of Edinburgh, Easter Bush Campus, Edinburgh, UK; 4 Land O’Lakes Venture37®, Arden Hills, MN USA; 5 Royal Jersey Agricultural & Horticultural Society (RJAHS), Trinity, Jersey Island Jersey; 6 Animal Breeding and Husbandry in the Tropics and Subtropics, University of Hohenheim, Stuttgart, Germany; 7 Scotlands’ Rural College (SRUC), Roslin Institute Building, Edinburgh, UK; 8 International Livestock Research Institute (ILRI), Nairobi, Kenya

**Keywords:** SNP arrays, Rwanda population structure, Admixture, Runs of homozygosity, Smallholder dairy

## Abstract

Previous genomic characterisation of Rwanda dairy cattle predominantly focused on the One Cow per Poor Family (locally called “Girinka”) programme. However, smallholder farmers in Rwanda have benefited from other livestock initiatives and development programmes. Capturing and documenting the genetic diversity, is critical in part as a key contribution to genomic resource required to support dairy development in Rwanda. A total of 2,229 crossbred animals located in all dairy-producing regions of Rwanda were sampled. For each animal, a hair sample was collected and genotyped by using the Geneseek Genomic Profiler (GGP, Neogen Geneseek^®^) Bovine 50 K (*n* = 1,917) and GGP Bovine 100 K arrays (*n* = 312). The combined dataset was subject to quality control, data curation for use in population genetics and genomic analyses. To assess the genetic structure and diversity of the current population, key analyses for population structure were applied: Principal Component Analysis (PCA), population structure and diversity, admixture analysis, measures of heterozygosity, runs of homozygosity (ROH) and minor allelic frequency (MAF). A dataset of global dairy population of European taurine, African indicus and African taurus (*n* = 250) was used as reference. Results showed that Rwanda cattle population is highly admixed of diverse pure and crossbred animals with average MAF of 33% (standard error; se = 0.001) with proportion of foreign high yielding (taurine) dairy breeds of Jersey Island (18%); 12% non-Island Jersey and 42% Holstein-Friesian ancestries. Two African *Bos taurus* and five *Bos indicus* breeds contributed 28% of their genetics. Genetic distances were highest in Gir and N’dama (0.29); and Nelore and N’dama (0.29). There were 1,331 ROH regions and average heterozygosity were high for Rwanda cattle (0.41 se = 0.001). Asides well-established genes in cattle, we found evidence for a variety of novel and less-known genes under selection to be associated with fertility, milk production, innate immunity and environmental adaptation. This observed diversity offers opportunity to decipher the presence and/or lack of genetic variations to initiate short- and long-term breed improvement programmes for adaptation traits, disease resistance, heat tolerance, productivity and profitability of smallholder dairy systems in Rwanda.

**Supplementary Information:**

The online version contains supplementary material available at 10.1186/s12863-025-01323-4.

## Introduction

Most livestock production systems in low- and middle-income countries rely on crossbred cattle derived from exotic and indigenous genetics to harness local adaptation traits of indigenous breeds and the high milk yield potential of exotic dairy breeds. North American and European *Bos taurus* dairy breeds, known for their high production levels, are routinely imported into Rwanda and other African countries for crossbreeding with indigenous breeds to improve productivity [1–3]. The utilisation of these crosses compensates for resilience to the tropical environment and disease susceptibility while maximising zootechnic (genetic) gains.

Most smallholder dairy systems in Africa are characterised by the use of poorly defined multi-generation genotypes of exotic and local breeds and managing fewer than ten dairy cows [4, 5]. Despite the overall low productivity compared to intensive systems, these smallholder farms are responsible for up to about 85% of the total milk produced within the region. For example, in East Africa, Holstein-Friesian genetics were imported for crossbreeding to Kenya, Uganda, Ethiopia, Rwanda and Tanzania [6–8] in the bid to optimise milk yield and income generation to smallholder households. The use of exotic purebred cows in Africa is expensive and therefore not cost-effective to smallholder farmers [9, 10]. To initiate and monitor genetic progress in smallholder systems, accurate performance data, pedigree recording and structured breeding programmes are lacking and impractical and therefore, an important gap that must be overcome to underpin breeding for genetic improvement. In addition, appropriate animal data identification, animal assignment to geographical locations for spatial modelling and herd connectedness to ascertain climate-smart and superior animals adapted to adverse changes in climate conditions are lacking in smallholder systems. A study by Selle et al. [11] tested the feasibility of utilising GPS coordinates from simulated and real data generated from smallholder dairy farms to enhance powerful spatial modelling for the identification of superior (climate-resilient) animals in smallholder systems [11]. This in part has the potential of overcoming some of the current challenges associated with low connectedness among farms which are also widely scattered and difficult to reach. Genomic based technologies provide a unique opportunity to optimise genetic gains and breeding potential of animals and hence reduce productivity gaps when phenotypes are limited or in some cases, unavailable [12, 13]. Previous studies have demonstrated the utilization of genomic predictions to counter constraints of near to impossible data availability [5, 14]. In developing economies (e.g. Africa) where livestock management systems are diverse and unstructured, smallholder systems could benefit from genomic-based prediction techniques as a useful alternative. Medium genotyping chips with 50 to 60 thousand single nucleotide polymorphisms (SNPs) have been utilised in global livestock populations for identification of SNPs related to; feed efficiency, animal longevity, survival, detection of genetic disorders, disease resistance, reproductive fitness, inbreeding coefficient, optimised genetic gains, accuracies of breed composition and genomic predictions for selection [15–17].

To match different genotypes to the diverse systems practiced, the knowledge of breed composition is paramount for the determination of which crossbreds perform best under the diverse variety of smallholder dairy systems and, as well as, which offspring with desired breed composition will succeed the next generation. Maximising the genetic potential of indigenous dairy breeds with crossbred animals of exotic genetics using genomic approach is a plausible strategy to optimise yield per cow. Therefore, selection of such productive breeds must strategically be based on monitoring genetic variation and inbreeding in the establishment of sustainable breeding programmes, accurate data collection and the utilisation of advanced technologies to develop genomic resources that support genetic improvement. Marshall et al. [18] highlighted the criteria for identification of appropriate crossbred genotypes for different livestock systems, breeding programmes and discovery of genetic variants of economic and ecological significance in Kenya, Senegal and Ethiopia.

Rwanda in particular, benefits from both national and non-governmental initiatives to promote farmer income and dairy productivity among smallholder households [19, 20]. A publication by Chagunda et al. [21] showing the genetic structure and diversity of dairy cattle under the Girinka programme demonstrated the potential of using such programme as a starting point for national breeding schemes. In the study, Chagunda et al. [21] applied high density SNP array (150 K) to 299 cattle from the Girinka programme to underpin the development of sustainable improvement strategies. Our study builds on Chagunda et al. [21] and aims to better understand how genomic analysis would assist in validating any dairy cattle sub-population through determination of population structure, genetic diversity, and breed proportions. Further, the study aimed at determining any barriers to genetic improvement such as inbreeding through runs of homozygosity that would culminate from non-divergent -sourced animals in different government- and non-governmental organisations-led initiatives.

## Materials and methods

### Ethical statement and approval

All procedures carried out in the study involving human (smallholder farmer) participants and procedure for animal hair sample collection were reviewed and approved by the Ethics Committee of the University of Rwanda’s Research and Postgraduate Studies (RPGS) unit in accordance with the 1964 Helsinki declaration and its later amendments or comparable ethical standards. Animal handling was done by trained technicians from Send A Cow Rwanda (SACR) and RAB to ensure adequate animal handling, and to minimise pain and injury to the animals during hair sample collection. Material Transfer Agreements (MTA) and the Nagoya Protocol on Access and Benefit Sharing (ABS) were signed among the following parties involved in the project namely; The University of Edinburgh on behalf of the Centre for Tropical Livestock Genetics and Health (CTLGH), The Royal Jersey Agricultural and Horticultural Society (RJAHS) and Rwanda Agriculture and Animal Resources Development Board (RAB). An import permit was obtained from the UK Department for Environment, Food and Rural Affairs (DEFRA) and a signed agreement for veterinary authorisation of sample collection in Rwanda and shipment to Neogen’s Dairy School in Ayr, Scotland, UK.

### Animal source and sampling

A total of 2,229 crossbred cattle were sampled from smallholder dairy farms (*n* = 1,110) previously selected to be part of the ‘Inka Nziza’ project and other national dairy initiatives [22]. These animals born from 2005 to 2020, are distributed in different agro-ecological regions and milk zones of Rwanda and are crossbreds of both indigenous (Ankole, Inkuku, Inkungu and Inyambo) and exotic cattle breeds (Holstein-Friesian, Jersey and Brown-Swiss). The regions include; Bugesera, Kayonza and Rwamagana of the Eastern province; Rulindo of the Northern province; Nyanza and Nyaruguru of the Southern province. The ‘Inka Nziza’ initiative was funded by Jersey Overseas Aid and implemented by the Royal Jersey Agricultural and Horticultural Society (RJAHS), RAB and SACR with the overall objective for dairy development and cattle genetic improvement in the Northern, Southern, Eastern and Western provinces of Rwanda. Hair samples were collected from the tail switch, taking proper care to avoid faecal contamination and adhering to a protocol provided by Neogen corporation.

### Reference dataset

A total reference dataset (*n* = 250) was built using existing data carefully selected to support analyses. A subset population (*n* = 204) of a panel of genotypes published by Bahbahani et al. [23] for commercial international taurine, indicine, and African taurine dairy breeds genotyped at high density (HD) were used as a reference for breed composition and diversity. The international taurine breeds were: Holstein and Jersey of West Europe (HOL; *n* = 25 and JER; *n* = 25). The African taurine breeds were: Ankole of Uganda (ANK; *n* = 25) and N’dama of Guinea (NDG; *n* = 24). The indicine breeds were: east African shorthorn zebu (EAZ_SH; *n* = 26) of Ethiopia; Gir of Brazil (GIR; *n* = 25); Nellore of Brazil (NEL; *n* = 25); Sahiwal of India (SHW; *n* = 13) and Sheko of Ethiopia (SHK; *n* = 16). To capture the impact and genetic proportion of the Jersey breed of Jersey Island origin (JER-JI), reference samples (*n* = 46) were provided by the Royal Jersey Agricultural and Horticultural Society who currently have off-springs born in Rwanda through artificial insemination or embryo transfer. To capture ancestry proportions and genetic signatures representative of African cattle, the African taurine breeds (NDG and ANK) and the five Indicine breeds (EAZ_SH, SHW, SHK, NEL and GIR) of Bahbahani et al. [24] were used. Total number of reference samples (*n* = 250) was added to our study dataset (2,229) resulting a total number of 2,479 animals used for further analyses.

### Genotyping and quality control

A population of the Rwanda crossbred cattle (*n* = 1,917) were genotyped for 47,843 SNPs using the Geneseek Genomic Profiler (GGP) 50 K SNP array, and an additional population of Rwanda crossbred cattle (*n* = 312) were genotyped for 95,256 SNPs using the GGP 100 K SNP array and mapped to the UMD3.1 bovine reference genome [25] for Illumina BovineHD Genotyping BeadChip^®^, respectively. Whereas the 204 samples from Bahbahani et al. [26] and Jersey Island samples (*n* = 46) corresponding to the breeds of interest for reference dataset of this study were genotyped for 777,962 SNPs and 47,843 SNPs, respectively. The SNP map of the UMD3.1 bovine reference genome was updated to the ARS-UCD 1.2 release so as to ascertain better knowledge of the cattle genome. That is, this was to, fix allele strand inconsistencies and lift-over of positions to the cattle reference ARS-UCD 1.2. The Allele AB strands were used across the reference, Rwanda samples and combined datasets to avoid mismatch of single base pairs, base pair positions and chromosome numbers for onward analyses.

All three datasets available were subject to quality control and data curation in R [26] and PLINK [27, 28] programmes. Quality control of genomic data was performed using the software PLINK [27, 28] considering the following exclusion criteria: non-autosomal SNP, SNP missing more than 10% of genotype data (individual call rate) and excluding SNPs missing more than 10% genotyping rate (marker call rate) and less than 5% minor allele frequency. Of our study dataset, 13 samples failed genotyping call rate of less than 90%. After data curation, 2,466 samples and 32,630 SNPs were remained for the downstream analyses.

### Minor allele frequencies (MAFs), hardy-weinberg principle (HWE) and heterozygosity

The SNPs were filtered in PLINK [27, 28] using the MAFs, Hardy–Weinberg equilibrium (HWE) and missing SNP proportions to remove SNPs with insufficient genotyping quality. Minor allele frequencies (MAFs) determined the degree of heterozygosity in each of the subpopulations (African taurine, African indicine, European taurine and Rwandan crossbred cattle. The SNP filtering based on the HWE was performed as we expected HWE deviations in the studied population due to sample size or genetic drift. The observed heterozygosity estimates for each population were calculated from observed genotype frequencies obtained from PLINK [27, 28]. Average expected heterozygosity (He) was assessed and observed heterozygosity (Ho) were averaged over loci by computing in PLINK under the assumption of HWE [27].

### Estimation of genetic diversity level, principal components and dimension reduction analyses

Using SNP variance-standardised relationship matrix for dimension reduction, the eigen values and eigen vectors generated from the PCA in PLINK were plotted and visualised using the “*tidyverse”* package and its dependencies [29] in R. Number of markers after principal component analyses was 32,630 SNPs. To validate the PCA, a weighted PCA (WPCA) was done by determining weights coefficient of the individual SNPs and genotypes for each of the individuals clustered by subpopulation [30]. In order to reduce the high dimensionality by preserving local relationships of the Rwanda population, Uniform Manifold Approximation and Projection; UMAP [31] was used to reduce the dimensionality with emphasis on fine-scale patterns between and within population groups. The WPCA and UMAP plots were visualised using “*weightedcluster*” and “*umap*” packages and their dependencies respectively in R [32, 33].

### Admixture and ancestry analyses

The SNPs of merged data, i.e. study and reference populations were curated (pruned) using PLINK [27, 28] to ensure that the individuals in the population, although admixed, were unrelated with no full-sibs or half-sibs. Therefore singletons SNP sites were excluded and linkage disquilibrium (LD) trimmed SNP sets were generated by removing one SNP from each pair of SNPs with R^2^ > 0.2 in 50 SNP blocks using PLINK v1.09b [28]. This implied that SNP that had an R^2^ value of greater than 0.2 with any other SNP within a 50-SNP sliding window (advanced by 2 SNPs each time) was removed. Therefore, 10,950 of 32,630 SNPs were removed due to high LD and 21,680 SNPs remained for 2,466 samples (i.e. 2,216 for Rwanda) and 10 reference breeds.

To determine the breed and ancestral proportion in the Rwanda population, ADMIXTURE program [34] was used to estimate the proportion of ancestry and breed introgression for each individual. ADMIXTURE program estimated the individual ancestry proportions given a *K* number of ancestral populations with maximum likelihood as well as identifying clusters to infer individual ancestries.

Supervised and unsupervised learning algorithms were employed while running admixture to 21,680 SNPs [34] for 2,466 samples and 10 reference breeds. To check for convergence of cross-validation iterations, the convergence parameters across runs were assessed by evaluating the increase in log-likelihood between iterations [35]. Eleven (11) independent runs with *k* ranging from *K* = 2 to *K* = 11 using the default parameters and cross-validation (CV) of 5-fold and then cross-validation of 10-fold were implemented so as to check changes in CV errors and ascertain the optimal number of clusters. For supervised learning, the population genetic structure assessment was performed in ADMIXTURE [34] to perform cross-validation iterations with a *k*-fold (K = 9) and the increase in log-likelihood between iterations. Supervised and unsupervised learning admixture outputs from ADMIXTURE were plotted and visualised as bar-plots using “*tidyverse”* package in R [36]. The best value of *k* for the learning methods was determined with a *k*-fold cross-validation clusters [37, 38] as thus;

$$\begin{aligned} \Pr\left(\left.\mathrm G\;\right|\;\mathrm K\right)=\mathrm Z\;\mathrm f\left(\left.\mathrm G\;\right|\;\mathrm Q,\;\mathrm P,\;\mathrm K\right)\;\mathrm\pi\left(\mathrm Q,\;\mathrm P\;\left|\;\mathrm K\right.\right)\;\mathrm{dQ}\;\mathrm{dP} \end{aligned}$$

### Pairwise Fst and phylogeny

The genetic differentiation among the populations and pairwise Fst values were calculated in PLINK [28] according to Wright’s formula [39, 40] taking into account sampling errors [41] and genetic differentiations between populations. The phylogeny analysis was carried out to ascertain evolutionary relationships between the populations. In order to ascertain the genetic distance and relationships for the subpopulations, the Nei’s genetic distance computed from Fst between and across-breed populations were constructed using neighbour-joining (NJ) relationship tree and then graphically displayed using “*vegan”* package in R [36].

### Runs of homozygosity (ROH), genomic inbreeding coefficient (FROH), functional enrichment analyses and phenotypic mapping of traits

An assessment of the ROH was conducted for identification of conserved genomic regions known to be generally common to cattle and other species. Consecutive runs [42] and minimal ROH length was set to 1,000 kb and a minimal of 30 SNPs (--homozyg-window-snp 30 and --homozyg-kb 1000) based on Mészáros et al. [16] and Biscarini et al. [43]. The default (1,000 kbps) minimum gap between consecutive SNPs was used, in order to account for the lower SNP density and SNP gap length in the 50 K SNP chip compared to the HD (~ 777 K) SNP chip [43]. The ROH regions were then used to compute genomic inbreeding coefficient (FROH) based on Bjelland et al. [44]. The ROH and FROH were visualised using the “*detectRUNS*” package in R [45].

The list of ROH genes were investigated on Ensembl Genes 86 database [46, 47] and the Ensembl BioMart tool on (http://useast.ensembl.org/biomart/martview/) using the bovine genome assembly ARS-UCD 1.2 [48]. The 1,331 ROH identified by the marker-based FST and ROH analyses were examined for genes (and their proteins functions) of biological significance using Protein ANalysis THrough Evolutionary Relationships (PANTHER) software version 14.0 [49]. To identify specific quantitative trait loci (QTL) and phenotype mapping of traits for bovine species, the base pair positions of samples and chromosomes with high values in the ROH for our study were submitted and verified in the cattle QTL database (CattleQTL^db^; release 47) [50]. The “*biomatr”* package in R was used to retrieve genes within the specific ROH regions [51] and the function of these genes were annotated at the NCBI website.

### Cross-breeding structure

In an attempt to understand the cross-breeding structure in the population studies, the proportion of breed composition were examined by year of birth. This presents how cross-breeding had evolved over time in the population and also presents the opportunity to help guide future direction. In addition, the breed composition of animals was examined by agro-ecological zones [52] to examine possible influence of climatic conditions and feed resources had influenced cross-breeding decisions. Finally, the relationship between herd size and the composition of cows reared was also examined.

## Results

Of the 2,229 animals genotyped from Rwanda, only 1,392 had birth dates corresponding to 872 herds and an average herd size was 1.60 (se = 0.91). There were 653 animals from Eastern province; Bugesera (*n* = 177); Kayonza (*n* = 235) and Rwamagana (*n* = 241). There were 235 animals from Northern province (Rulindo; *n* = 239) and 500 animals from Southern province; Nyanza (*n* = 252) and Nyaruguru (*n* = 248).

Total number of samples before genomic data analyses and combining the datasets derived from the 2 chips resulted in 43,765 SNPs (*n* = 2,479 animals) across the breed populations. After data curation, 2,466 samples and 21,680 SNPs were remaining for admixture evaluation after linkage disequilibrium (LD), principal component analyses (PCA) and other subsequent analyses.

### Herd size and estimated population genetic diversity

**Fig. 1:**
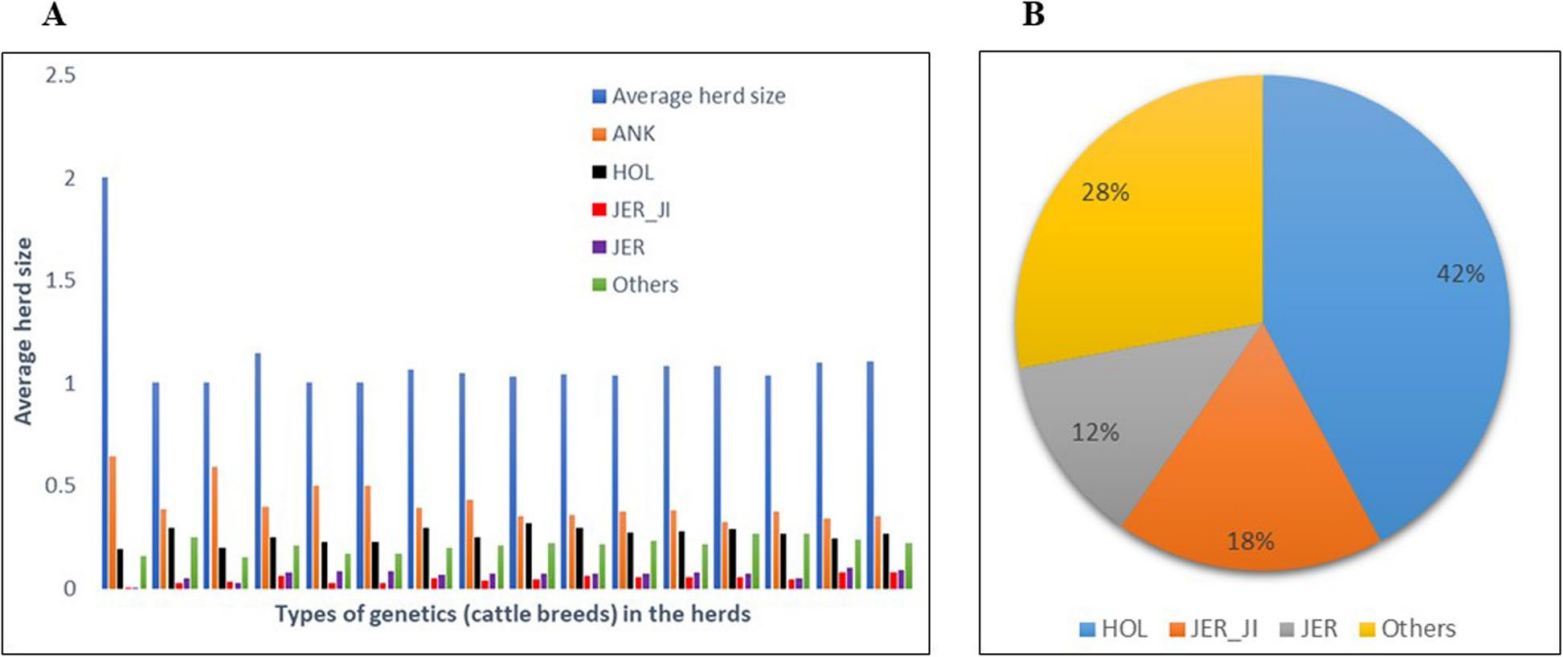
Relationship between herd size and breed composition of animals (**A**) and percentage distribution of foreign high yielding (exotic) dairy and indigenous breeds to the Rwanda population (**B**)

The study population had higher levels of contribution from local Ankole as the main indigenous breed used for crossbreeding compared to other Indicine breeds in Rwanda (Fig. 1A). The average proportion of exotic Jersey Island genes in the population was 18% (± 0.01%) while the greater percentage of 42% was of Holstein (HOL) of West Europe, 12% non-Island Jersey ancestries (of West Europe) as well as 28% contributions from other breeds (5% east African shorthorn zebu of Ethiopia, 3% Gir of Brazil, 3% Nellore of Brazil, 7% Ankole of Uganda, 3% N’dama of Guinea, 4% Sahiwal of India and 3% Sheko of Ethiopia). The 18% estimated JER_JI contribution from our study originated from the ‘Inka Nziza’ project on behalf of the Island of Jersey. The Rwanda dairy population exhibited varying degrees of proportions of foreign high yielding (exotic) dairy breeds due to recent cross breeding (Fig. 1B).

The average heterozygosity estimates were highest for the Rwanda cattle (0.42 ± 0.001) and lowest and the same for Gir and N’Dama breeds (0.21 ± 0.001 and 0.21 ± 0.001), respectively). Heterozygosity estimates for European taurine breeds used as references ranged from 0.34 ± 0.001 for JER; 0.38 ± 0.001 for JER_JI; 0.39 ± 0.001 for HOL. For African indicines, heterozygosity ranged between 0.27 ± 0.001 and 0.32 ± 0.001. Average MAF largely driven by the proportion of SNPs ranged from 0.33% (± 0.001) for Rwanda population and 34% (± 0.001) across the merged (Rwanda dairy population vs. reference population) population (Table 1).

**Table 1:** Sample frequency, minor allele frequency, expected and observed heterozygosity of the studied Rwanda population (mean ± standard error; se)

Breed(s)	*n*	H_e_	se	H_o_	se	MAF	se
Ankole (ANK)	25	0.31	0.001	0.32	0.001	0.24	0.001
East African shorthorn zebu (EAZ_SH)	26	0.31	0.001	0.31	0.001	0.23	0.001
Holstein-Friesian (HOL)	25	0.39	0.001	0.41	0.001	0.32	0.001
Island Jersey (JER_JI)	46	0.38	0.001	0.39	0.001	0.29	0.001
non-Island Jersey (JER)	25	0.34	0.001	0.35	0.001	0.26	0.001
Gir (GIR)	25	0.21	0.001	0.21	0.001	0.15	0.001
Nellore (NEL)	25	0.21	0.001	0.21	0.001	0.15	0.001
N’dama (NDG)	24	0.27	0.001	0.27	0.001	0.20	0.001
Sahiwal (SHW)	13	0.30	0.001	0.30	0.001	0.22	0.001
Sheko (SHK)	16	0.32	0.001	0.32	0.001	0.23	0.001
Rwanda (RWA)	2,216	0.42	0.001	0.41	0.001	0.33	0.001
Merged curated dataset	2,466	0.43	0.001	0.41	0.001	0.34	0.001

*n* sample size, *H*_*e*_ expected heterozygosity, *H*_*o*_ observed heterozygosity, *MAF* minor allelic frequency, *ANK* Ankole, *EAZ_SH* east African shorthorn zebu, *GIR* Gir, *HOL* Holstein, *JER* non-Island Jersey, *JER_JI* Island Jersey, *NDG* N’dama, *NEL* Nellore, *RWA* Rwanda, *SHK* Sheko, *SHW* Sahiwal

### Population genetic structure, admixture and ancestry

**Fig. 2:**
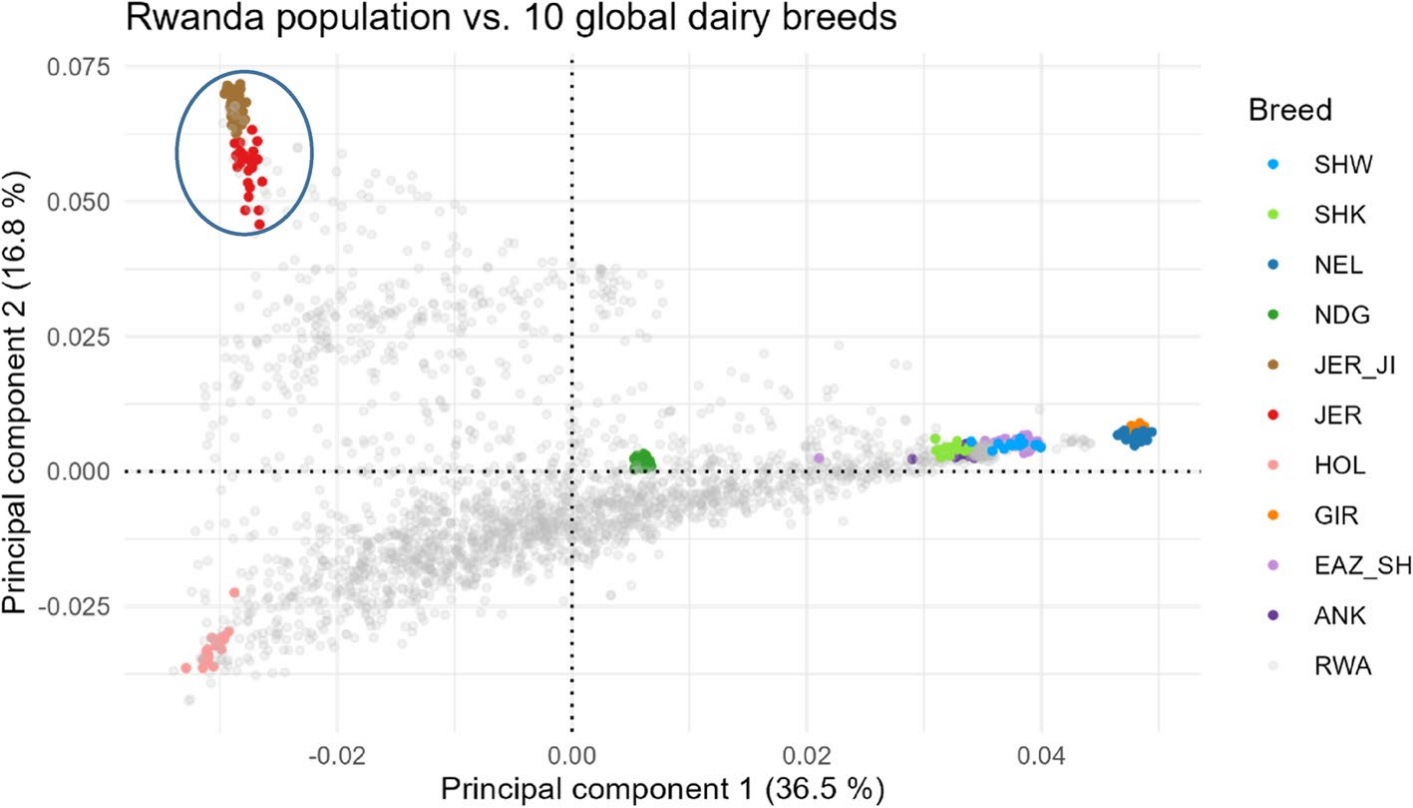
Principal component analysis of Rwanda cattle vs. global reference population

Our results from principal components and admixture analyses showed that the Rwanda cattle population is a highly admixed (crossbred) population with European taurine breeds such Holstein and Jersey (West Europe); Jersey (Jersey Island) and African indicine and Zebu (Sahiwal, Ankole) representing the number of different origins or cluster that can be defined from the genetic data (K = 9, 10 & 11). The PCA for Rwanda population vs. global reference showed large variation. The first principal coordinate vector accounted for 36.5% of total variation with a significant contribution of European breed (HOL), African *Bos indicus* (NDG), and separated the GIR and NEL breeds. The Rwandan animals dispersed evenly between ANK, SHW, SHK and east African shorthorn zebu (EAZ_SH) and to a lower extent; GIR and NEL breeds. The second principal coordinate vector accounted for 16.8% of the total variation and separated the Island and non-Island Jersey breeds highlighted in the blue circle (Fig. 2).

**Fig. 3:**
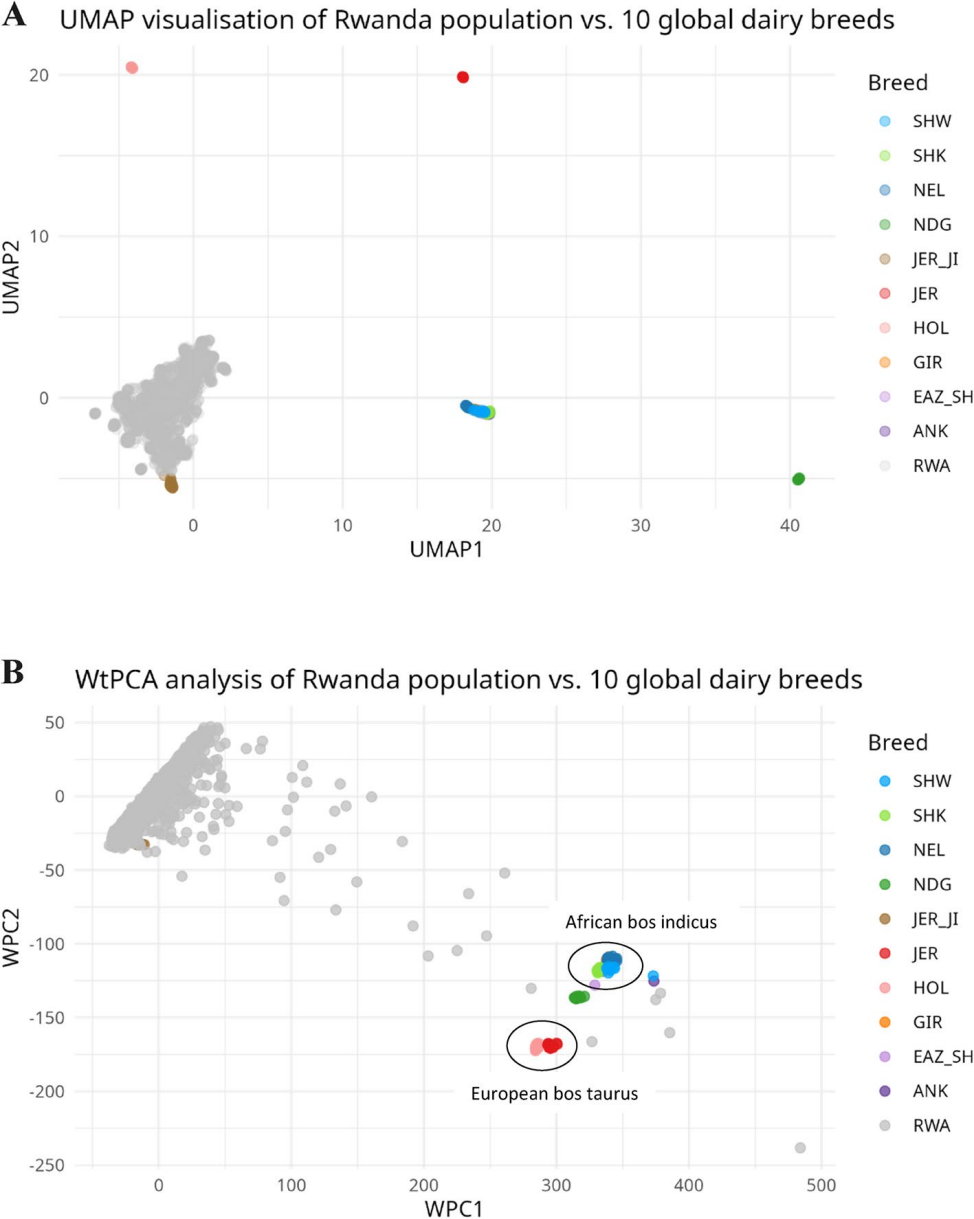
UMAP presentation plot of Rwanda cattle vs. global reference population (**A**) and weighted principal component analysis (WPCA) of Rwanda cattle vs. global reference population (**B**). **A** and **B** provides a more detailed representation of diversity and relationships among the studied populations when compared to the conventional PCA plot in Fig. 2

The UMAP and weighted PCA plots revealed a reduced dimensionality and distinctiveness highlighting a distinct fine scaling of the Rwanda population from the European taurine and African indicine breeds (Fig. 3) when compared to the PCA plot in Fig. 2.

**Fig. 4:**
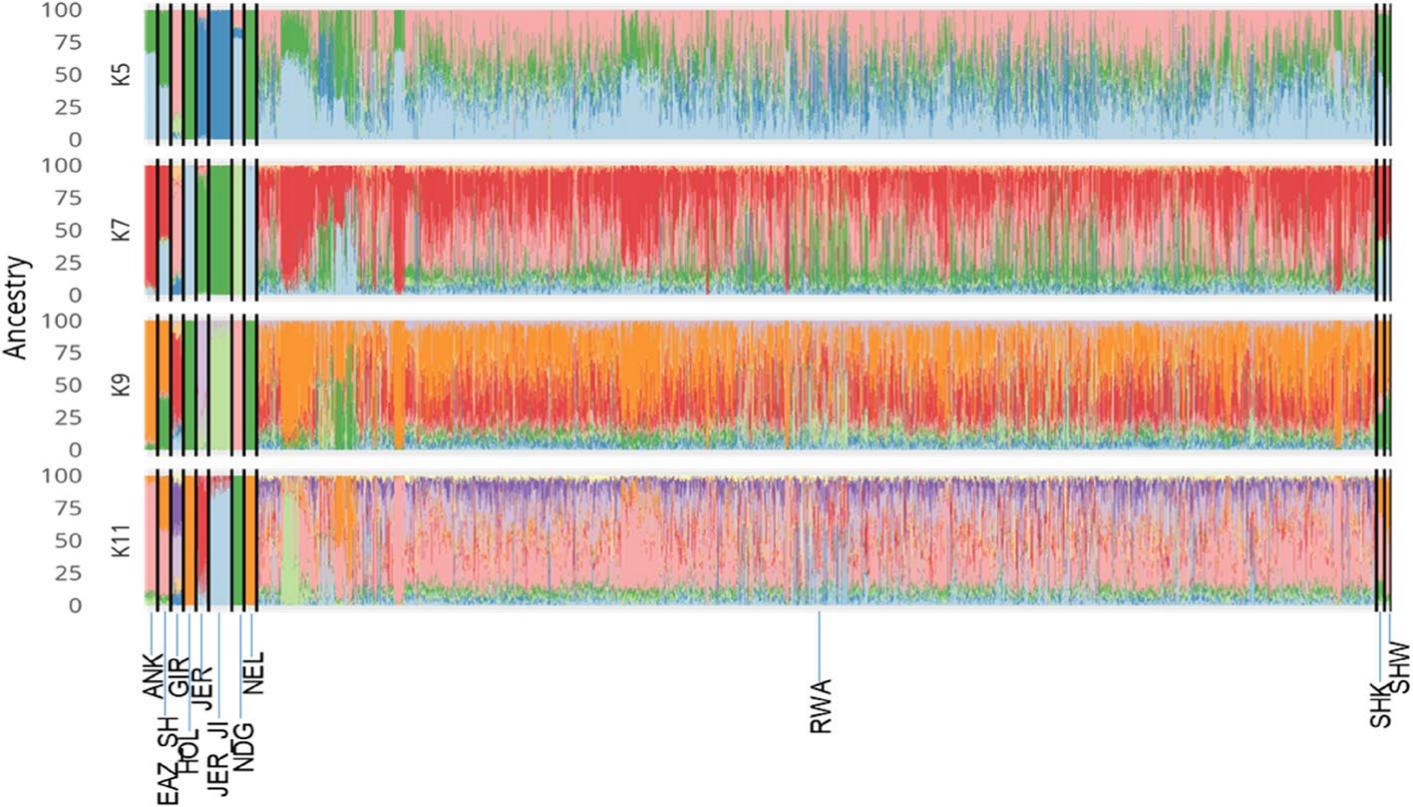
Admixture bar plots showing breed proportions and introgression at selected and assumed ancestry assignment clusters K (5, 7, 9 and 11). Each horizontal bar from left to right, represents Ankole (ANK), east African shorthorn zebu (EAZ_SH), Gir (GIR), Holstein (HOL), non-Island Jersey (JER), Island Jersey (Jersey-JI), N’dama (NDG), Nellore (NEL), Rwanda (RWA), Sheko (SHK) and Sahiwal (SHW). The proportion of the bar in each of the *k* cluster colours corresponds to the average posterior likelihood that the individual is assigned to the cluster indicated by that colour

The cross validation (CV) errors for both unsupervised and supervised Admixture learning ranged between 0.59 and 0.65, respectively for all the breeds represented in the study irrespective of inclusion or exclusion of GIR and NEL breeds. Each line bar of the Admixture plot is an individual partitioned by breed (Fig. 4) and each breed population is separated by black lines. For unsupervised learning, the K value which is the number of the subpopulation that makes up the total population was at K10 (CV = 0.60) and K11 (CV = 0.59). But based on scrutiny of each CV errors, visual inspection of the admixture and PCA plots, K = 9 represented the most appropriate population number for the studied dataset. Importantly, increasing K above 9 did not reveal any detectable population substructure and the breed clusters remained the same.

The K10 and K11 with NEL and GIR was 0.60. Although not reported here, excluding both breeds had lower but same CV at K10 and K11, respectively (0.61; 0.60). For supervised learning with or without GIR and NEL, CV were higher than unsupervised learning procedures (K9 = 0.65; K11 = 0.64).

### Pairwise fixation index, phylogeny and genetic distance between populations

The mean value for the genetic (Fst) distance for 21,680 SNP markers and weight genetic (Fst) of all the pairwise comparison of the breeds was the same (0.12 ± 0.0004). Genetic distances between populations were highest in Gir and N’dama and Nelore and N’dama breeds (0.29). Large differentiations were observed between the indicine and taurine breeds (Table 2).

**Table 2:** Pairwise genetic differentiation statistic (Fst values; upper diagonal) among study populations

Population	RWA	Jer_JI	ANK	EAZ_SH	GIR	HOL	JER	NDG	NEL	SHK	SHW
RWA	0	0.09	0.041	0.053	0.130	0.047	0.081	0.109	0.133	0.046	0.023
JER_JI		0	0.174	0.181	*0.254*	0.148	0.07	0.206	*0.256*	0.175	0.185
ANK			0	0.031	0.148	0.147	0.168	0.146	0.1496	0.032	0.037
EAZ_SH				0	0.09	0.157	0.175	0.176	0.093	0.019	0.004
GIR					0	*0.251*	*0.262*	*0.294*	0.059	0.134	0.103
HOL						0	0.129	0.188	*0.252*	0.253	0.148
JER							0	0.207	*0.265*	0.165	0.175
NDG								0	*0.293*	0.136	0.189
NEL									0	0.136	0.106
SHK										0	0.023
SHW											0

*ANK* Ankole, *EAZ_SH* east African shorthorn zebu, *GIR* Gir, *HOL* Holstein, *JER* non-Island Jersey, *JER_JI* Island Jersey, *NDG* N’dama, *NEL* Nellore, *RWA* Rwanda, *SHK* Sheko, *SHW* Sahiwal. Italicised values depicts substantial differentiation ranging from 0.25 to 0.29 observed in the Indicine breed (GIR) and Taurine breeds

**Fig. 5:**
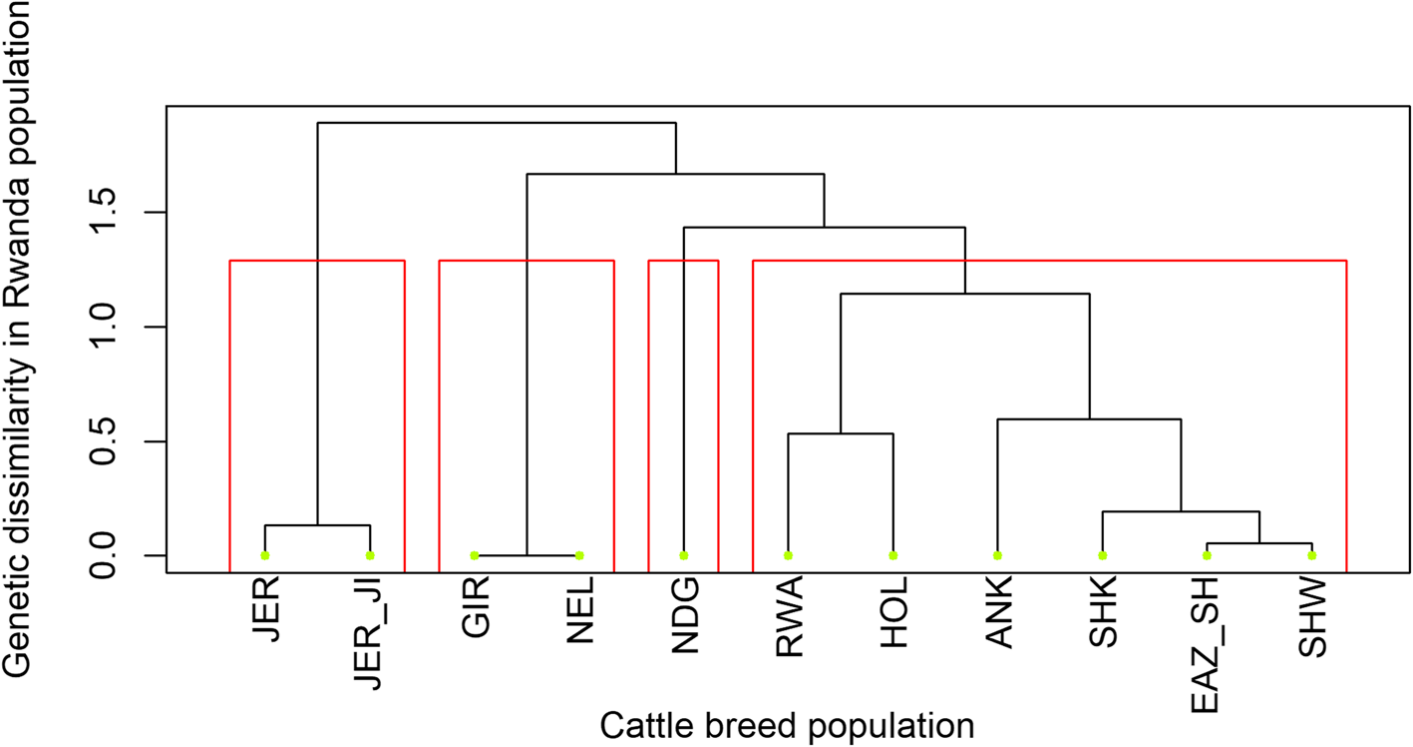
Phylogenetic tree showing relationships between reference populations and Rwanda cattle. Breeds are labelled as; Ankole (ANK), east African shorthorn zebu (EAZ_SH), Gir (GIR), Holstein (HOL), non-Island Jersey (JER), Island Jersey (JER_JI), N’dama (NDG), Nellore (NEL), Rwanda (RWA), Sheko (SHK) and Sahiwal (SHW). The red boxes illustrate clusters or subpopulations of cattle breeds represented in the studied population from Rwanda

The Rwanda population showed close and diverse genetic relationships with Holstein-Friesian breed than across the other reference populations. This implies a substantial amount of crossbreeding at every level in the population (Fig. 5).

### Runs of homozygosity (ROH) and inbreeding coefficient

Analyses of the studied population identified 1,331 ROH regions in 785 individuals with an average number of SNP markers in run (180.0 ± 3.10). The length of the run ranged between 1,006 and 61,947 kilobases, and an average length of 7,030.0 ± 145.53 kilobases. The average proportions of detected homozygosity and heterozygosity within the same ROH were 0.97 ± 0.001 and 0.01 ± 0.0001, respectively. The genomic inbreeding coefficient (FROH) across the genome of the studied populations ranged from 0.0004 to 0.05 with an average FROH of 0.005 ± 0.0001 (Table 3).

**Table 3:** Average genomic inbreeding coefficient for the runs of homozygosity of the studied Rwanda population (mean ± se)

Group	Individuals in RoH	Mean FRoH per breed
ANK	14	0.002 ± 0.0004
EAZ_SH	9	0.04 ± 0.003
GIR	19	0.005 ± 0.001
HOL	18	0.01 ± 0.001
JER	23	0.008 ± 0.001
JER_JI	46	0.03 ± 0.001
NDG	12	0.003 ± 0.0009
NEL	19	0.005 ± 0.0008
RWA	619	0.04 ± 0.0002
SHK	4	0.001 ± 0.0007
SHW	2	0.001 ± 0.000

*ANK* Ankole, *EAZ_SH* east African shorthorn zebu, *GIR* Gir, *HOL* Holstein, *JER* non-Island Jersey, *JER_JI* Island Jersey, *NDG* N’dama, *NEL* Nellore, *RWA* Rwanda, *SHK* Sheko, *SHW* Sahiwal

**Fig. 6:**
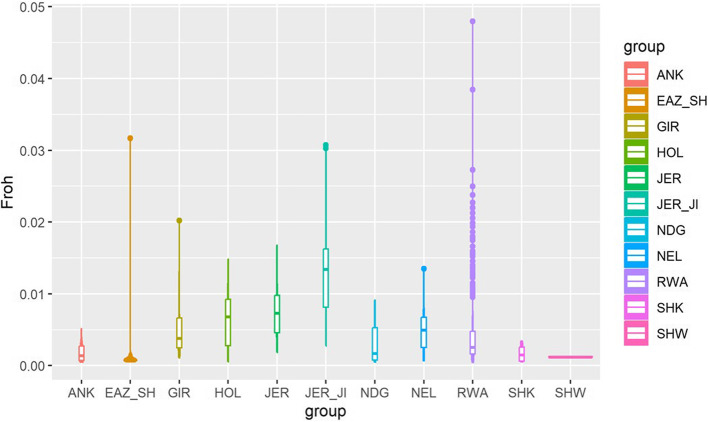
Violin plot showing genomic inbreeding coefficient detected for the populations where each coloured violin represents a cattle population

The mean number of runs of homozygosity per individual population were few in Sahiwal (*n* = 2) and highest in the Rwanda population (*n* = 619). Per breed, the mean FROH ranged from 0.001 (SHW and SHK) to 0.04 (RWA and EAZ_SH). The average FROH value was highest in our study population in Rwanda; RWA (0.04 ± 0.0001), followed by Island Jersey; JER_JI (0.03 ± 0.0001) and lowest in Ankole, (0.001 ± 0.0005); Sahiwal (0.001 ± 0.000) and Sheko (0.001 ± 0.0007). The colours for the violin plot reflect inbreeding coefficient values based on detected ROH for the breeds as indicated in the (right) legend in Fig. 6.

**Fig. 7:**
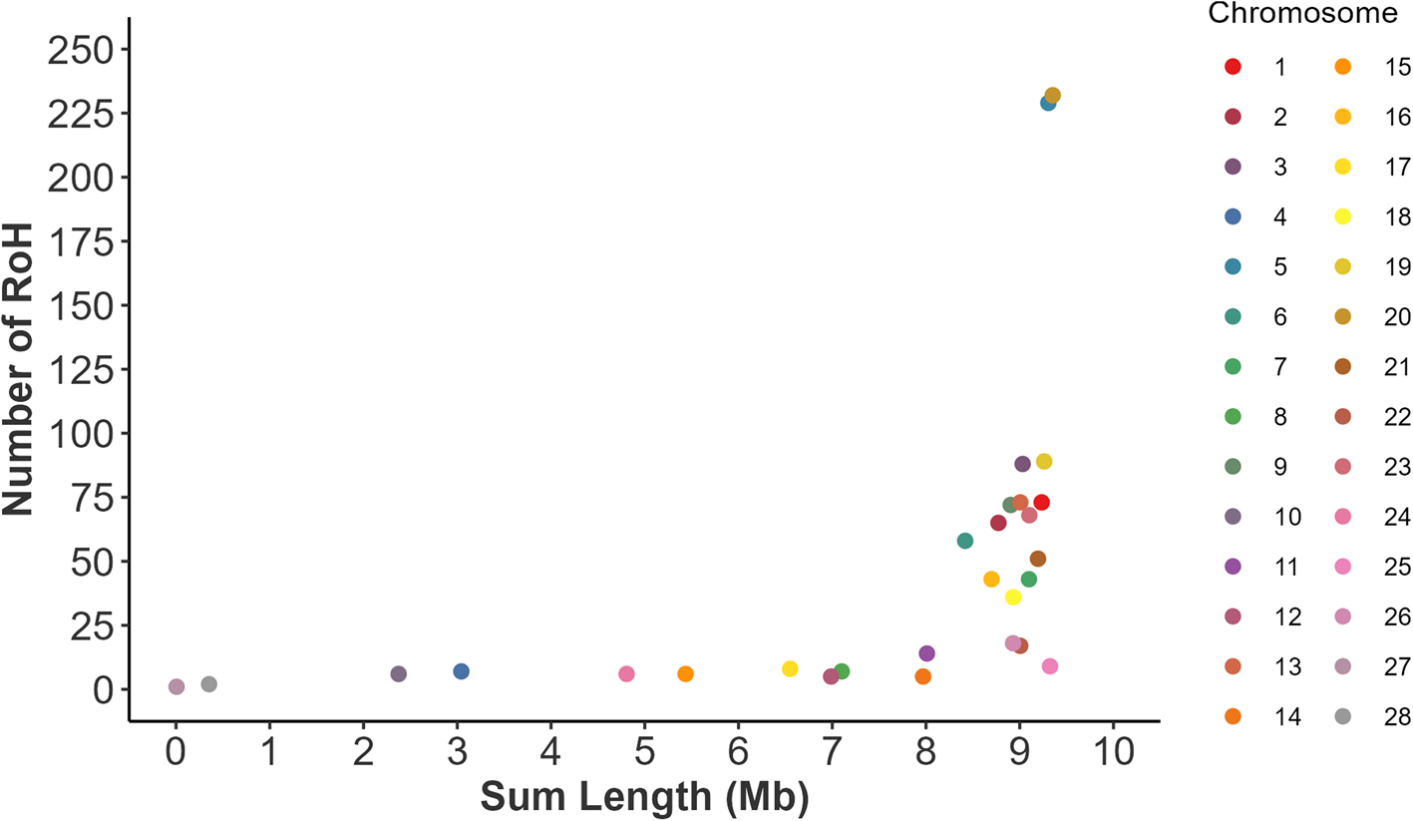
Sum length of ROH (in mega bases) across the chromosomes in studied population. High peaks and higher sum length (in mega bases) at chromosomes 5 and 20 can be observed in the ROH regions

Chromosomes 5 and 20 have respectively 229 (213 individuals) and 232 (206 individuals) ROHs of the total 1,331 ROH estimated for the population. Chromosome 5 and 20 had the highest number of ROH measured across the chromosome and sum of SNP length. High peaks and higher sum length (in mega bases) of the specific SNPs in the 1,331 ROH were observed (Fig. 7) while particular peaks in genomic positions were observed on chromosomes 5 and 20 featured in ROH which were shared in approximately 50% of the sampled animals.

**Fig. 8:**
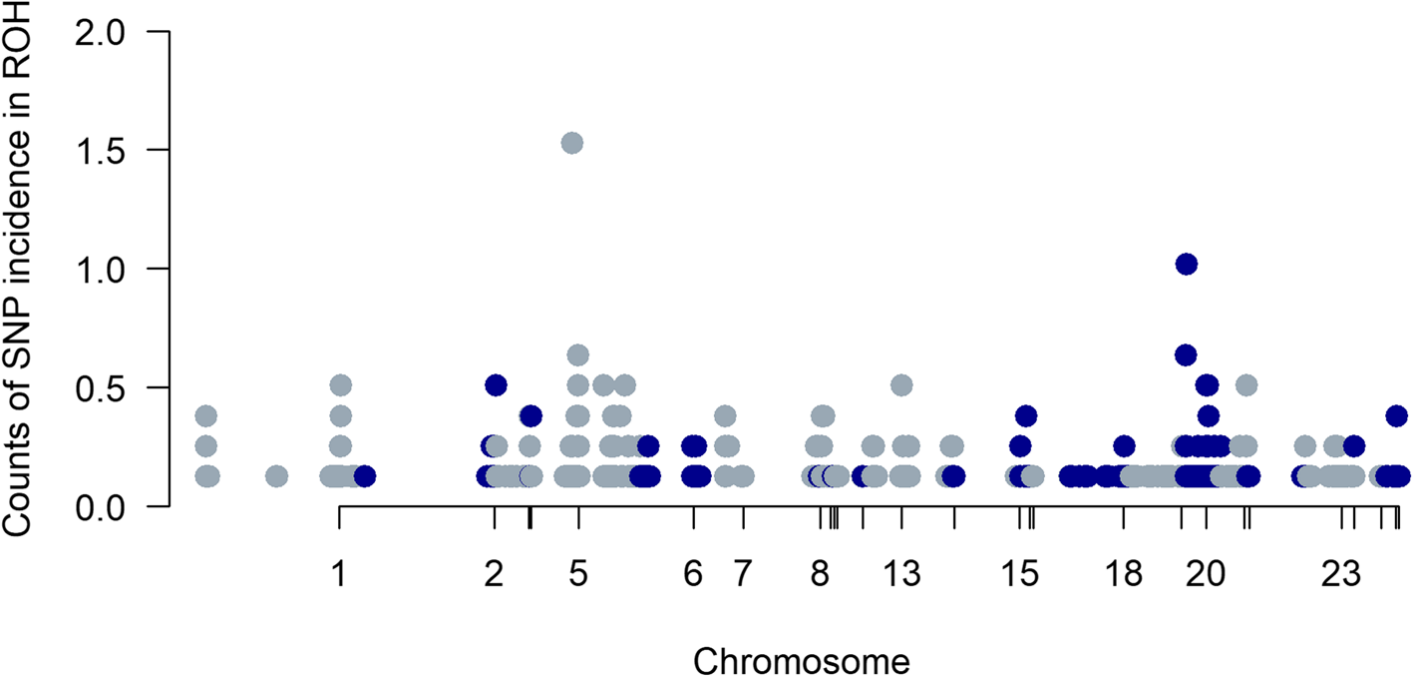
Manhattan plot of counts of SNPs occurrences of a SNP by chromosomes in ROHs across individuals in the population

High peaks for proportion of SNP occurrence in ROH were observed at chromosomes 5 and 20. Figure 8 shows Manhattan plots based on percentage of animals with specific SNPs in the 1,331 ROH in the studied population.

### Mapping of ROH genes of biological importance in cattle

Of the 1,331 ROH regions, several cattle genes matched the ARS-UCD 1.2 bovine platform. For the PANTHER classification analyses, the genes matched the *Bos taurus* and other species. Biological significance of the homozygosity association results identified multiple genes on chromosomes 5 and 20. These genes appeared to be mainly involved in biological processes, molecular function and cellular components. Major biological processes involved include; behaviour, growth, immune system regulation, metabolic process, response to stimulus, reproduction and reproduction processes. Higher proportions of genes were involved in cellular and metabolic functions and as well as biological regulations and response to stimulus. Based on molecular functions, higher proportions of the ROH genes were involved in binding and catalytic activities as well as molecular transducer and transcription regulator activities.

From the cattleQTL^db^, the regions for chromosome 5 and 20 have been mapped to be associated with phenotypic traits of global importance in cattle. A total of 82 unique cattle genes in the 1,331 ROH regions were found in chromosomes 5 (*n* = 53 genes) and 20 (*n* = 29 genes). Some of genes in the ROH are well-established genes reported in literature for chromosomes 5 and 20. However, we observed a variety of less-known (novel) genes under selection to be associated with fertility, milk production, innate immunity and environmental adaptation These genes include; *AVIL*, *B4GALNT1*, *NEMP1*, *SNORA62*, *TAC3* and *ZBTB39* (chromosome 5); and *EFCAB9*, *GABRP*, *INSYN2B*, *MIR218 - 2* and *MIR103 A1* (chromosome 20). Also, 382 and 504 QTLs have been reportedly associated with chromosomes 5 and 20, respectively. Table 4 shows the ROH base pair regions between 54,884,085 and 56,860,046 (chromosome 5), between 263,956 and 4,684,304 (chromosome 20) and their reported traits and QTLs in cattle.

**Table 4:** Reported association studies and quantitative trait loci (QTL) for chromosomes 5 and 20 in the RoH

	Breeds	Traits	Genes identified in ROH	Reported data	No. of QTLs
Chromosome 5	Blonde d'aquitaine, Angus and Holstein	Calving ease	SHMT2; GLI1; NEMP1^*^	QTL and Association	13
	Fleckvieh, Tropical composite and Canchim	Coat colour and heat tolerance	DCTN2; MYO1 A	Association	13
	Unknown	Interval to first oestrus after calving	CDK4; NAB2	Association	12
	Holstein, Tropical composite	Milk fat yield	DDIT3; B4GALNT1^*^; SNORA62^*^	QTL	13
	Holstein, Hereford, Tropical composite	Immune system regulation and adaptation	bta-mir- 2430; bta-mir- 2431; bta-mir- 677; CYP27B1; GLI1; KIF5 A; MBD6	QTL and Association	13
	Tropical composite	Inhibin level	INHBC; INHBE	QTL and Association	131
	Ayrshire and Holstein	Milk yield	ATP5 F1B; LAP3; CTDSP2	QTL	17
	Canchim	Scrotal circumference	CDK4	Association	46
Chromosome 20	Charolais, Gelbvieh, Hereford, Limousin, Simmental, Angus and Hereford	Metabolic body weight	ERGIC1; PANK3; SH3PXD2B; HSD17B6	Association	175
	Charolais, Gelbvieh, Hereford, Limousin, Simmental and Angus	Average daily gain	PANK3	Association	39
	Angus, Gelbvieh, Nanyang, Hereford, Charolais, Limousin and Simmental	Body (birth and growth) weight	ERGIC1, NPM1	QTL and Association	38
	Angus, Charolais, Gelbvieh, Hereford, Limousin and Simmental	Carcass weight	DOCK2; KCNIP1; STK10	QTL and Association	37
	Tropical composite, Nelore and European cattle breeds	Immune system regulation and adaptation	LCP2, FOXI1	QTL and Association	14
	Tropical composite, Vrindavani and European cattle breeds	Coat colour	*RANBP17*	QTL and Association	16
	Blonde d'aquitaine and Holstein	Calving ease (maternal)	FGF18; KCNMB1	Association and QTL	15
	Blonde d'aquitaine and Holstein	Spermatogenesis (sperm motility)	EFCAB9^*^; FBXW11; SPZ1	Association and QTL	14
	Charolais, Gelbvieh, Hereford, Limousin, Simmental, Angus and Tropical composite	Dry matter intake/rumen metabolism	bta-mir- 12032; NEURL1B	Association	30

*QTL* quantitative trait loci

^*^less reported genes under selection in our study

### Cross-breeding structure

**Fig. 9:**
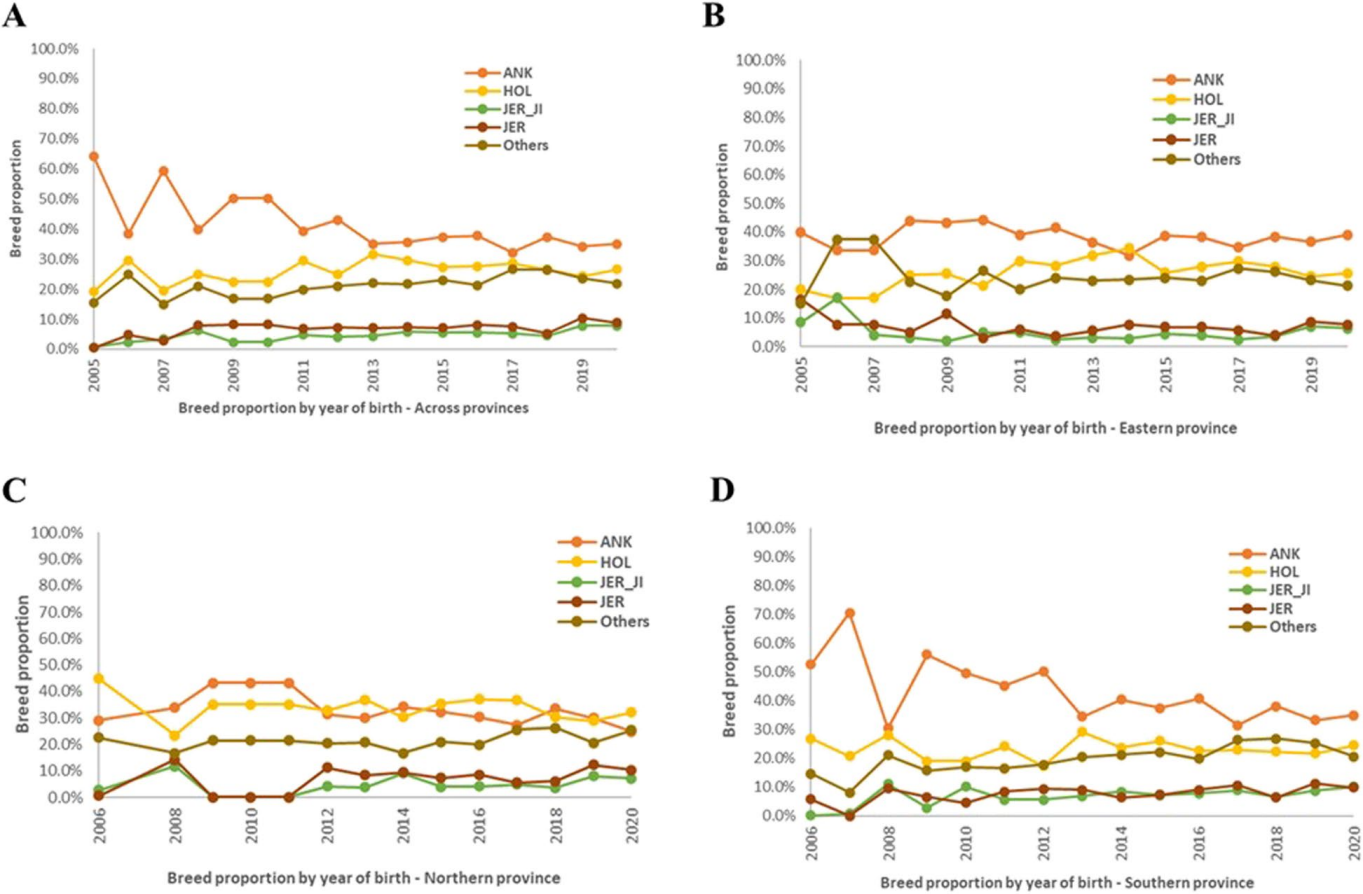
Evolution of crossbreeding over 15-year (2005–2020) period across the reported provinces in Rwanda (**A**). Ankole breed was mainly used for crossbreeding with Holstein-Friesian, Jersey breeds than with other breeds in the Eastern (**B**), Northern (**C**) and Southern (**D**) provinces

The utilization of Holstein-Friesian as the main exotic breed followed by the Jersey breeds were predominantly similar within each province. Figure 9 A shows the average composition of animals by year of birth while Fig. 9B, C and D shows the average composition of animals by different agroecological regions. The results in Fig. 9A indicates that across the area studied, cross-breeding seem to have been maintained at a level to ensure a good balance of exotic and indigenous genetics with farmers aiming to increase productivity while maintaining adaptive capacity of animals.

Similarly, Fig. 9C indicates that the crossbreeding structure is similar in both Eastern and Southern provinces but lower levels of ANK have been maintained in the Northern province. The herd sizes are rather too small to identify any pattern between herd size and the type of animals kept but it appears that cows with higher proportion of ANK, HOL and Other breeds are preferred in most dairy farm herds. These Other breeds include; east African Zebu shorthorn, N’dama, Sheko, Sahiwal, Gir and Nellore breeds.

## Discussion

This study provides valuable information for assessing the current genetic diversity and genetic structure of Rwanda’s dairy cattle population, and to support further development and use of genomic tools to improve the dairy sector. Principal component and admixture analyses confirmed Rwanda cattle as a highly admixed (crossbred) population. Our study shows that of the 2,229 Rwanda cattle sampled, the highest contribution of exotic genetics is of Holstein breed (HOL; 42%), followed by Jersey breed (Jer_JI: 18%; JER: 12%) and other breeds of indigenous origin (28%). The principal component analyses indicates that the admix population observed in our study is similar to other dairy populations in Africa and therefore, this is further discussed later. Genes of interest were detected in the runs of homozygosity (ROH) regions that could further be studied. We have also demonstrated the usefulness of evaluating ROH regions for estimating inbreeding when pedigree is lacking; an approach which could be utilized in mating plans in future development of the dairy systems in Rwanda. We anticipated that the genotyped animals would guide future genomic approaches for directional selection for on-farm productivity, genetic progress, healthy and feed-efficient animals that best adapt to the diverse production systems and tropical environment in Africa.

The genetic diversity assessment of Rwanda’s dairy population reveals attempts to breed cattle through crossbreeding for dairy genetics that is suitable for the local production systems. The main target has been the utilisation of exotic breeds to upgrade productivity of the indigenous cattle. Crossbreeding in Rwanda dairy industry has been characterised by the use of mainly Holstein-Friesian [21], Ankole and the Jersey breed. The Holstein-Friesian and Jersey are among the exotic dairy breeds used extensively in pure and crossbreeding in the tropics [53].

Rwanda is supported by various dairy for development projects (as a means to improving milk yield and income) through the diversification of a crossbred dairy population. The genetic contributions of Holstein breed (HOL; 42%) and non-Island Jersey (JER; 12%) most likely originated from beneficiaries of Heifer International, International Fund for Agricultural Development and Girinka programmes [19]. The Jersey_JI (18%) in our study originated from the Jersey Island through artificial insemination and embryo transfer procedures. Genetic contributions of N’dama, Gir and European *Bos taurus* breeds were previously reported in Rwanda [21]. Moderately to significantly highest heterozygosity (0.41 ± 0.001) was observed in our study (RWA: 0.41); European taurines (HOL: 0.41; JER: 0.35; JER_JI: 0.39), African taurine (NDG: 0.27); east African shorthorn zebu (EAZ: 0.31) and Indicine breeds (ANK: 0.32; GIR: 0.21) than in a previous study in Rwanda under Girinka dairy programme (study cattle: 0.38; Holstein: 0.38; Jersey: 0.30; east African shorthorn zebu: 0.26 and Gir: 0.18). Similarly, heterozygosity for our study population were higher than estimates reported by Cheruiyot et al. [54] in Tanzania (Holstein: 0.37; JER: 0.31; N’dama: 0.25 and east African shorthorn zebu: 0.28). However, same estimates as our study for Gir breed (0.21) was observed in the same study of Cheruiyot et al. [54]. From the principal component analysis, the dispersal of Rwanda animals to the GIR and NEL breed, suggests a contribution of the two breeds albeit to a lower extent. In addition, the Uniform manifold approximation and projection (UMAP) and weighted PCA plots revealed a reduced dimensionality suggestive of a distinct fine scaling of the Rwanda population from the European taurine and African indicine breeds. The UMAP and weighted PCA have been adopted in visualising unique cluster patterns and population structure in humans and other species [31, 55, 56].

The highest contribution of breeds to Rwanda cattle were from Holstein and Jersey genetic components. The local Ankole was the main indigenous breed used for crossbreeding with Holstein-Friesian to enhance dairy productivity across the provinces and districts. The local Ankole has low milk production with an average milk yield of 1.33 to 4.58 L/day [57]. When improved with Holstein genetics and properly managed, they are more efficient and produce greater yield of about 5 to 10 L/day [19]. Milk production in Rwanda dairy systems is heavily dependent on the availability of feed resources and water [20] even with the presence of appropriate genetics. There are 6 major agro-ecological zones (AEZ) in Rwanda [52] and influencing factors for dairy productivity in each of these zones include; temperature, altitude, rainfall, topography, crop production, livestock population, soil type and weather variability [58]. The Eastern province has an average annual temperature of 22.53ºC (72.55ºF) and it is 2.09% higher than Rwanda’s averages (www.weatherandclimate.com). For instance, the Eastern province is one of the major dairy producing region in Rwanda with sufficient availability of rainfall and pasture/forage for grazing favouring milk yield. Previous studies by Manzi et al. [59] showed that Ankole and Holstein-Friesian crossbred cows reared in Eastern AEZ had the highest average milk yield compared to Western and Central AEZ. The Southern province has an annual temperature of 20.51ºC (68.92ºF) and it is 0.07% higher than Rwanda’s averages (www.weatherandclimate.com). The Southern province is prone to seasonal drought [60] with pasture/forage shortage and therefore cows depend on communal dams or rivers as their major drinking water source [61]. The Northern province has a tropical type of climate, characterized by successions in rainy and drought seasons offering a favourable climate for farming [62]. From our study, the Northern province seem to prefer cows with slightly higher exotic genes. In general, the Northern province has an average temperature of about 16.22ºC (61.2ºF) and it is − 4.22% lower than Rwanda’s averages (www.weatherandclimate.com). These temperatures may be more favourable for cows with higher exotic genes. Temperature extremes resulting to heat stress is a major concern for livestock especially for those managed in tropics. In our study, we were not able to assess temperature, humidity or weather data to underpin comprehensive assessments and differences in heat stress days and temperature rise in the AEZs.

The dominance of Holstein genetics for East African crossbred cattle has been reported in previous studies in Ethiopia [63]; Uganda [64]; Kenya [65]; Tanzania [54] and Rwanda [21]. Similar findings have been reported in North African [4] and West African crossbred cattle [66]. The dispersal patterns (i.e. breed introgression) observed in this study generally reflects farmer’s efforts in upgrading animals to high exotic genetic levels in a bid to increase productivity. The existence of genomic tools has widely shifted the landscape for selecting animals for dairy cattle breeding with the aim to improve performance of purebred animals in developed countries and as well as crossbred cattle [9]. Microsatellite markers and SNPs distributed all over the genome have been used for genetic characterisation of different livestock species; e.g. cattle [67], goats [68–70], sheep [71–73], pigs [74], chickens [75, 76], camels [77, 78] and horses [79, 80]. With the existence of genomic information, the effects of inbreeding have been estimated by using homozygosity runs and genomic inbreeding coefficients as an alternative to pedigree inbreeding where pedigree data are scarce. However, a combination of both genomic information (genotype) and pedigree information (phenotypes) allows the opportunity to develop and implement methods to manage populations at the genomic level and as well as positively altering any sustainable breeding programmes. Runs of homozygosity has been widely used in livestock species for signals of genotype-phenotype association and phenotypes of interest [81].

The inbreeding coefficient observed in the Rwanda population was very low, less than 1% (0.04 ± 0.0002) and a highest heterozygosity of 41% (0.41 ± 0.001). Lower detectable levels for genomic inbreeding has been reported in Tanzanian crossbreds (i.e. Lushoto cattle; 0.033 standard deviation (SD) 0.03 and Rungwe cattle: 0.02 SD 0.04) [52]. Wiggans, et al. [82] found average inbreeding of 4.7% in Ayrshire cows, 3.0% in Guernsey, 2.6% in Holstein, 3.3% in Jersey, and 3.0% in the Brown Swiss breed using pedigree relationships. Unfavourable genomic estimates per 1% increase in genomic-based inbreeding have been reportedly comparable or slightly larger than pedigree-based estimates [44, 81, 83]. Estimating the inbreeding percentage for potential mating helps to minimise the risks of inbreeding and recessive conditions in dairy herds. In the UK, inbreeding levels for breeds was about 2% in 2012 and is gradually increasing by 0.13% annually [84]; but also, significantly below the 6% recorded in the United States of America [84]. All exotic dairy cattle breeds are genetically small populations with limited number of bulls used for artificial insemination. For instance, the Holstein breed, like other temperate dairy breeds have a limited genetic size at the global level because of the extensive utilisation of the North American germplasm in the 1980 s [85]. Studies have also shown such genetic influence of North American germplasm to European, French and British dairy cattle breeds [86–89]. The low genetic size generates an inbreeding increase rate of approximately 1% per generation and is the leading cause of low genetic merits and recurrent emergences of recessive defects [87]. As this national figure rises, it will generally impact negatively on performance and more genetic defects will be imminent. In financial terms, a 1% increase in inbreeding costs results in a loss of 34 kg of milk per lactation, reduction in 13.1 days of productive life and a £14.11 loss in lifetime net income [84].

Globally, the acceptable level of genomic inbreeding for dairy cattle herd is 6.25% [90]. However, international inbreeding levels are increasing and is being monitored [85, 90] and an important goal for future dairy breeding programmes [83]. In the UK, inbreeding levels are now higher to almost 8% as a result of breeding of close relatives, parentage misidentification and selection for specific traits [91] leading to widespread utilisation of genetically related individuals as parents of the next generation. Therefore, the dairy industry experts recommend that farmers control inbreeding as much as possible and avoid threshold levels higher than 6.25% [85, 91].

Our study implied that inbreeding is currently not a challenge for Rwanda’s dairy cattle population. The population studied for Rwanda has recently experienced an admixture of Island Jersey genetics under the ‘Inka Nziza initiative through the use of artificial insemination and embryo transfer. Therefore, it would be of importance to sample this population for any changes in FROH estimates to inform future genomic improvement strategies as the effective (dairy) population size in Rwanda increases. In addition, it will be of importance to monitor inbreeding in future breed improvement programmes. The FROH can be used to accurately assess individual inbreeding levels compared to other inbreeding coefficient estimators [92–94].

We identified greater than 50 ROH regions at chromosomes 1, 3, 5, 9, 13, 19 and 20. However, significant and prominent ROH regions and genes were associated with chromosomes 5 and 20. Aside’s well-established candidate genes of economic importance reported in literature [65, 80], we also identified less-known genes which could be linked with fertility, coat colour and adaptation, innate immune process, and milk yield. These genes include; AVIL, B4GALNT1, EEF1 AKMT3, NAB2, NEMP1, SNORA62, TAC3 and ZBTB39 for BTA5 and EFCAB9, GABRP, INSYN2B, MIR103 A1, MIR218 - 2 and SPDL1 for BTA20. The ROH genes and chromosomes identified from our study have been reported to be associated with multiple functions in both dairy and beef cattle (for example [95–98]),. Chromosomal ROH regions associated with both production and fertility traits have been identified for *Bos taurus* autosome (BTA) 1, 13 and 19 [99] and in BTA 8, 13, 14 and 19 [100]. Similarly, Biscarini et al. [43] also used ROH to detect genomic regions observed on BTA 3, 5, 7, 13 and 18 known to be associated with susceptibility to overlapping disorders; infectious, metabolic, respiratory, reproductive, locomotive diseases and mastitis in dairy cows under intensive farming conditions. Furthermore, ROH region on BTA19 revealed that when homozygous, had an adverse effect on milk production traits [43]. It has been noted that signatures of selection proximate to BTA 19 region have implicated growth hormone gene 1 (GH1) as a potential candidate gene that encodes the growth hormone binding the growth hormone receptor. GH1 is therefore a promising candidate gene marker for improving fertility [97], growth [101], meat [102] and milk production [96] in cattle. Also, Huson et al. [103] identified multiple genes of biological significance for immune regulation and metabolic processes in chromosomes 5, 24 and 27 in the Island Jersey than non-Island Jersey cattle. The Rwanda population in our study have crossbreds with the Jersey breed where 30% of breed proportion originates from the Jersey Island. Further investigation of regions in the genome of crossbred tropical (indigenous) x Jersey Island cattle will be useful for downstream analyses and future investigation of immune regulation and metabolic processes for genomic selection of tropically adapted crossbred cattle for low-input systems.

The *Bos taurus* (BTA) 20 is empirically known to be associated to the slick phenotype (PRLR gene) for the short hair coat of Senepol and Carora cattle [95]. The slick hair gene is considered to be directly associated with higher thermo-tolerance and indirectly with important production trait, as it is consistently associated with improved production traits in crossbreds under tropical environment [104–106]. The BTA 20 is reportedly associated with higher milk yield [107], milk composition [108], fertility [108], maternal calving ease [109], growth [109] and clinical mastitis disease [108]. Similarly, Pryce et al. [81] also found a ROH region on BTA20 in both Holstein and Jersey cattle. Other studies have revealed the mutations of slick gene is associated with hairy syndrome, excessive coat length and severe lactation dysfunction in cattle [110]. Further, we identified *RANBP17* on BTA20 responsible for coat colour and *MYO1 A* gene on BTA5 responsible for coat colour and heat tolerance. The genes have been reported in previous studies [111, 112]. A study by Yin and König [113] identified candidate genes on BTA5 to be associated with maternal body weight in German Holstein dairy cows. Body weight at any stage of a cow’s development is of utmost importance in dairy breeding schemes due to their strong correlations with feed energy efficiency and their impact on longevity, cow health and farm economics.

By exploring the potential of both pure and crossbred animals in Rwanda, there is potential to select for resilient, productive and ultimately profitable animals by utilisation of genomic resources and genomic-related tools. Studies have demonstrated the possibilities of selection signatures for adaptation traits, disease tolerance, parentage assignment, inbreed levels, variation in milk yield, conservation strategies and accurate estimation of breed composition [17, 24, 114, 115] in cattle in Africa in order to inform future selection of desirable breed traits. The utilisation of genomic information in our study provided insights to the current genetic make-up of Rwanda’s dairy cattle population in the current dairy farming systems. The Jersey breed irrespective of the origin showed a diversity of its use within Rwanda. Therefore, we propose the use of genomic approaches for the selection of superior on productivity traits which could close the productivity gap. Alongside closing productivity gaps, challenges around animal welfare, herd health, disease resilience and thermo-tolerance could be mitigated to maximise productive and reproductive performance in the cattle population. Also, additional data on location to understand/decipher the agro-ecologies of the smallholder systems could be useful in assigning different dairy ecotypes to the diverse systems of production.

This study contributes and provides a comprehensive view of the crossbreeding structure in Rwanda over time and in the different regions. The current animals generally have a good blend of exotic and indigenous breeds even in different regions. Milk records are being collected in the ongoing study and the cross-breeding structure will be valuable in evaluating phenotypic and genetic trends in milk productivity over time. Hence, such information will assist in the next stage of designing cross breeding strategies to optimise productivity and adaptability in the country.

## Conclusion

Our study assessed the current genetics of Rwanda’s crossbred dairy population as well as regions of interest that could help inform future precision breeding techniques where pedigree information are lacking. The cross-breeding structure indicate a good blend of exotic and indigenous breeds to optimise productivity and adaptation with some slight regional differences. The identified genes could be used as target genes for future marker-assisted selection. The admixture results will therefore be valuable in evaluating the right breed mix for different regions as production data becomes available. While there is direct relationship between herd size and the breed composition of animals kept, farmers seem to prefer cows with higher proportion of ANK, HOL and Others. The information from this study provides a good frame work to design the next stages of cross breeding in Rwanda.

## Application

This study contributes to a better understanding of the genetic architecture of Rwanda dairy cattle population that could best enhance and drive tropical dairy improvement strategies through genomic selection. The population studied provides the platform for the training of individuals for subsequent collation of genomic and phenotypic data to enable future genomic selection. While the study provides insights to the sustainable application of genomics as a tool that underpins livestock adaptability to climate change and the availability of animal based sourced foods, it is also critical to monitor and maintain the diversity of locally adapted indigenous cattle breeds to Rwanda, East Africa and the tropics so as to prevent diversity losses.

## Supplementary Information

Supplementary Material 1.

## Data Availability

The data that supports the findings of this study are available on request from the corresponding author.
