# Supplementary material for: The genetic structure and diversity of smallholder dairy cattle in Rwanda
Source: BMC Genom Data. 2025 May 27;26:38. doi: 10.1186/s12863-025-01323-4 (PMC12107919; doi:10.1186/s12863-025-01323-4)
Supplement: Supplementary file 1 — Supplementary Material 1. [file 12863_2025_1323_MOESM1_ESM.docx]

**Table 1**. Sample frequency, minor allele frequency, expected and observed heterozygosity of the studied Rwanda population (mean±standard error; se).

| Breed(s) | n | H_e_ | se | H_o_ | se | MAF | se |
| --- | --- | --- | --- | --- | --- | --- | --- |
| Ankole (ANK) | 25 | 0.31 | 0.001 | 0.32 | 0.001 | 0.24 | 0.001 |
| East African shorthorn zebu (EAZ_SH) | 26 | 0.31 | 0.001 | 0.31 | 0.001 | 0.23 | 0.001 |
| Holstein-Friesian (HOL) | 25 | 0.39 | 0.001 | 0.41 | 0.001 | 0.32 | 0.001 |
| Island Jersey (JER_JI) | 46 | 0.38 | 0.001 | 0.39 | 0.001 | 0.29 | 0.001 |
| non-Island Jersey (JER) | 25 | 0.34 | 0.001 | 0.35 | 0.001 | 0.26 | 0.001 |
| Gir (GIR) | 25 | 0.21 | 0.001 | 0.21 | 0.001 | 0.15 | 0.001 |
| Nellore (NEL) | 25 | 0.21 | 0.001 | 0.21 | 0.001 | 0.15 | 0.001 |
| N’dama (NDG) | 24 | 0.27 | 0.001 | 0.27 | 0.001 | 0.20 | 0.001 |
| Sahiwal (SHW) | 13 | 0.30 | 0.001 | 0.30 | 0.001 | 0.22 | 0.001 |
| Sheko (SHK) | 16 | 0.32 | 0.001 | 0.32 | 0.001 | 0.23 | 0.001 |
| Rwanda (RWA) | 2,216 | 0.42 | 0.001 | 0.41 | 0.001 | 0.33 | 0.001 |
| Merged curated dataset | 2,466 | 0.43 | 0.001 | 0.41 | 0.001 | 0.34 | 0.001 |

n (sample size); H_e_ (expected heterozygosity); H_o_ (observed heterozygosity); MAF (minor allelic frequency);

Ankole (ANK), east African shorthorn zebu (EAZ_SH), Gir (GIR), Holstein (HOL), non-Island Jersey (JER), Island Jersey (JER_JI), N’dama (NDG), Nellore (NEL), Rwanda (RWA) and Sheko (SHK).

**Table 2**. Pairwise genetic differentiation statistic (Fst values; upper diagonal) among study populations.

| Population | RWA | Jer_JI | ANK | EAZ_SH | GIR | HOL | JER | NDG | NEL | SHK | SHW |
| --- | --- | --- | --- | --- | --- | --- | --- | --- | --- | --- | --- |
| RWA | 0 | 0.09 | 0.041 | 0.053 | 0.130 | 0.047 | 0.081 | 0.109 | 0.133 | 0.046 | 0.023 |
| JER_JI |  | 0 | 0.174 | 0.181 | 0.254 | 0.148 | 0.07 | 0.206 | 0.256 | 0.175 | 0.185 |
| ANK |  |  | 0 | 0.031 | 0.148 | 0.147 | 0.168 | 0.146 | 0.1496 | 0.032 | 0.037 |
| EAZ_SH |  |  |  | 0 | 0.09 | 0.157 | 0.175 | 0.176 | 0.093 | 0.019 | 0.004 |
| GIR |  |  |  |  | 0 | 0.251 | 0.262 | 0.294 | 0.059 | 0.134 | 0.103 |
| HOL |  |  |  |  |  | 0 | 0.129 | 0.188 | 0.252 | 0.253 | 0.148 |
| JER |  |  |  |  |  |  | 0 | 0.207 | 0.265 | 0.165 | 0.175 |
| NDG |  |  |  |  |  |  |  | 0 | 0.293 | 0.136 | 0.189 |
| NEL |  |  |  |  |  |  |  |  | 0 | 0.136 | 0.106 |
| SHK |  |  |  |  |  |  |  |  |  | 0 | 0.023 |
| SHW |  |  |  |  |  |  |  |  |  |  | 0 |

Ankole (ANK), east African shorthorn zebu (EAZ_SH), Gir (GIR), Holstein (HOL), non-Island Jersey (JER), Island Jersey (JER_JI), N’dama (NDG), Nellore (NEL), Rwanda (RWA), Sheko (SHK) and Sahiwal (SHW).

**Table 3**. Average genomic inbreeding coefficient for the Runs of homozygosity of the studied Rwanda population (mean±se).

| Group | Individuals in RoH | Mean FRoH per breed |
| --- | --- | --- |
| ANK | 14 | 0.002±0.0004 |
| EAZ_SH | 9 | 0.04±0.003 |
| GIR | 19 | 0.005±0.001 |
| HOL | 18 | 0.01±0.001 |
| JER | 23 | 0.008±0.001 |
| JER_JI | 46 | 0.03±0.001 |
| NDG | 12 | 0.003±0.0009 |
| NEL | 19 | 0.005±0.0008 |
| RWA | 619 | 0.04±0.0002 |
| SHK | 4 | 0.001±0.0007 |
| SHW | 2 | 0.001±0.000 |

Ankole (ANK), east African shorthorn zebu (EAZ_SH), Gir (GIR), Holstein (HOL), non-Island Jersey (JER), Island Jersey (JER_JI), N’dama (NDG), Nellore (NEL), Rwanda (RWA) and Sheko (SHK).

**Table 4**. Reported association studies and quantitative trait loci (QTL) for chromosomes 5 and 20 in the RoH.

| Chromosome 5 | Breeds | Traits | Genes identified in ROH | Reported data | No. of QTLs |
| --- | --- | --- | --- | --- | --- |
|  | Blonde d'aquitaine, Angus and Holstein | Calving ease | SHMT2; GLI1; NEMP1* | QTL and Association | 13 |
|  | Fleckvieh, Tropical composite and Canchim | Coat colour and heat tolerance | DCTN2; MYO1A | Association | 13 |
|  | Unknown | Interval to first oestrus after calving | CDK4; NAB2 | Association | 12 |
|  | Holstein, Tropical composite | Milk fat yield | DDIT3; B4GALNT1*; SNORA62* | QTL | 13 |
|  | Holstein, Hereford, Tropical composite | Immune system regulation and adaptation | bta-mir-2430; bta-mir-2431; bta-mir-677; CYP27B1; GLI1; KIF5A; MBD6 | QTL and Association | 13 |
|  | Tropical composite | Inhibin level | INHBC; INHBE | QTL and Association | 131 |
|  | Ayrshire and Holstein | Milk yield | ATP5F1B; LAP3; CTDSP2 | QTL | 17 |
|  | Canchim | Scrotal circumference | CDK4 | Association | 46 |
| Chromosome 20 | Charolais, Gelbvieh, Hereford, Limousin, Simmental, Angus and Hereford | Metabolic body weight | ERGIC1; PANK3;  SH3PXD2B; HSD17B6 | Association | 175 |
|  | Charolais, Gelbvieh, Hereford, Limousin, Simmental and Angus | Average daily gain | PANK3 | Association | 39 |
|  | Angus, Gelbvieh, Nanyang, Hereford, Charolais, Limousin and Simmental | Body (birth and growth) weight | ERGIC1, NPM1 | QTL and Association | 38 |
|  | Angus, Charolais, Gelbvieh, Hereford, Limousin and Simmental | Carcass weight | DOCK2; KCNIP1; STK10 | QTL and Association | 37 |
|  | Tropical composite, Nelore and European cattle breeds | Immune system regulation and adaptation | LCP2, FOXI1 | QTL and Association | 14 |
|  | Tropical composite, Vrindavani and European cattle breeds | Coat colour | *RANBP17* | QTL and Association | 16 |
|  | Blonde d'aquitaine and Holstein | Calving ease (maternal) | FGF18; KCNMB1 | Association and QTL | 15 |
|  | Blonde d'aquitaine and Holstein | Spermatogenesis (sperm motility) | EFCAB9*; FBXW11; SPZ1 | Association and QTL | 14 |
|  | Charolais, Gelbvieh, Hereford, Limousin, Simmental, Angus and Tropical composite | Dry matter intake/ rumen metabolism | bta-mir-12032; NEURL1B | Association | 30 |

QTL: quantitative trait loci; *less reported genes under selection in our study
